# Food insecurity and food consumption by season in households with children in an Arctic city: a cross-sectional study

**DOI:** 10.1186/s12889-017-4393-6

**Published:** 2017-06-15

**Authors:** Catherine Huet, James D. Ford, Victoria L. Edge, Jamal Shirley, Nia King, IHACC Research Team, Lea Berrang-Ford, Shuaib Lwasa, Didacus Namanya, Cesar Carcamo, Alejandro Llanos, James D. Ford, Victoria L. Edge, Sherilee L. Harper, Sherilee L. Harper

**Affiliations:** 1 Department of Geography, McGill University, Montreal, QC H3A OB9 Canada; 2 Office of the Chief Science Officer, Public Health Agency of Canada, Guelph, ON N1G 5B2 Canada; 3 Nunavut Research Institute, Nunavut Arctic College, Iqaluit, NU XOA OHO Canada; 4 Department of Population Medicine, University of Guelph, Guelph, ON N1G 2W1 Canada

**Keywords:** Food security, Food preparation, Food consumption, Season, Socioeconomic status, Indigenous, Inuit, Children, Nunavut, Iqaluit

## Abstract

**Background:**

High rates of food insecurity are documented among Inuit households in Canada; however, data on food insecurity prevalence and seasonality for Inuit households with children are lacking, especially in city centres. This project: (1) compared food consumption patterns for households with and without children, (2) compared the prevalence of food insecurity for households with and without children, (3) compared food consumption patterns and food insecurity prevalence between seasons, and (4) identified factors associated with food insecurity in households with children in Iqaluit, Nunavut, Canada.

**Methods:**

Randomly selected households were surveyed in Iqaluit in September 2012 and May 2013. Household food security status was determined using an adapted United States Department of Agriculture Household Food Security Survey Module. Univariable logistic regressions were used to examine unconditional associations between food security status and demographics, socioeconomics, frequency of food consumption, and method of food preparation in households with children by season.

**Results:**

Households with children (*n* = 431) and without children (*n* = 468) participated in the survey. Food insecurity was identified in 32.9% (95% CI: 28.5–37.4%) of households with children; this was significantly higher than in households without children (23.2%, 95% CI: 19.4–27.1%). The prevalence of household food insecurity did not significantly differ by season. Demographic and socioeconomic characteristics of the person responsible for food preparation, including low formal education attainment (OR_Sept_ = 4.3, 95% CI: 2.3–8.0; OR_May_ = 3.2, 95% CI: 1.8–5.8), unemployment (OR_Sept_ = 1.1, 95% CI: 1.1–1.3; OR_May_ = 1.3, 95% CI: 1.1–1.5), and Inuit identity (OR_Sept_ = 8.9, 95% CI: 3.4–23.5; OR_May_ = 21.8, 95% CI: 6.6–72.4), were associated with increased odds of food insecurity in households with children. Fruit and vegetable consumption (OR_Sept_ = 0.4, 95% CI: 0.2–0.8; OR_May_ = 0.5, 95% CI: 0.2–0.9), as well as eating cooked (OR_Sept_ = 0.5, 95% CI: 0.3–1.0; OR_May_ = 0.5, 95% CI: 0.3–0.9) and raw (OR_Sept_ = 1.7, 95% CI: 0.9–3.0; OR_May_ = 1.8, 95% CI: 1.0–3.1) fish were associated with decreased odds of food insecurity among households with children, while eating frozen meat and/or fish (OR_Sept_ = 2.6, 95% CI: 1.4–5.0; OR_May_ = 2.0, 95% CI: 1.1–3.7) was associated with increased odds of food insecurity.

**Conclusions:**

Food insecurity is high among households with children in Iqaluit. Despite the partial subsistence livelihoods of many Inuit in the city, we found no seasonal differences in food security and food consumption for households with children. Interventions aiming to decrease food insecurity in these households should consider food consumption habits, and the reported demographic and socioeconomic determinants of food insecurity.

**Electronic supplementary material:**

The online version of this article (doi:10.1186/s12889-017-4393-6) contains supplementary material, which is available to authorized users.

## Background

Food insecure households face challenges affording or obtaining sufficient and nutritious food for an active and healthy life [1]. Approximately 11% of Canadian households were classified as being food insecure in 2011–2012 [2]. In contrast, 63% of Inuit households in Arctic Canada were classified as being food insecure [3, 4], and community-based surveys have indicated an even higher prevalence in some Inuit communities [5–7]. This high prevalence spurred research examining the determinants, distribution, and experiences of food insecurity in the North, which has highlighted the complexity of Northern food systems [5, 6, 8, 9]. For instance, Inuit diet is traditionally comprised of nutrient-dense “country” foods [10–12] (i.e. country foods are ‘animals and plants harvested from the local environment’ (11) that are not typically available at retail stores, hereafter referred to as “local foods”) such as caribou, seal, and fish. However, Inuit diet has undergone a nutrition transition whereby retail foods are now widely consumed and are often a main food source [11–15]. The poor nutritional quality of many retail foods that are available in the North increases the risk of nutritional deficiencies [11, 13, 16]; furthermore, the high cost of these foods, mainly due to their transport [17–22], can impact households’ food security status, particularly when local foods are not readily available [11].

Food insecurity is often associated with inadequate nutrient intakes and lower diet quality, which can compromise adults’ [22–24] and children’s [3, 25–30] health and well-being. For instance, food insecurity can have detrimental long-term effects on child physical, mental, cognitive, and psychosocial health and development [22, 26, 30–36]. As such, Arctic food security research has increasingly focused on households with children: high household food insecurity was documented among Inuit preschoolers aged 3–5 years in Nunavut [7] and Inuit children aged 3–14 years in Nunavik (Northern Quebec) [34]. Responding to this high food insecurity prevalence has recently emerged as a priority for governments. For instance, the Nunavut Food Security Coalition, co-chaired by the Government of Nunavut and Nunavut Tunngavik Incorporated, released the Nunavut Food Security Strategy and Action Plan (NFSSAP) in May 2014, which identifies food security among households with children as a public health concern [31] and charts a strategy to address the high levels of food insecurity in Nunavut [37, 38].

While research on food security in households with children is burgeoning, seasonality of food security with regards to children has scarcely been investigated in the global literature, and, to our knowledge, has not been published for Inuit communities. Season has an important influence on Inuit food systems since access to, and success of hunting, trapping, and fishing depend on season-dependent factors including ice conditions, precipitation patterns, and animal migration and distribution [37, 39–42]. As a result, the type and quantity of local food harvested and consumed in Inuit communities typically changes seasonally [11, 16, 43]. Furthermore, weather conditions also affect retail food transportation into communities [37, 42, 44]. Recent research in Iqaluit found no significant difference in household food security status between seasons [45]; however, seasonal differences in food security for households with children have not been investigated.

Furthermore, food security research in larger northern centres is nascent; past studies focused on households with children in small northern communities (<2000 people) [5, 6, 8, 9, 46] or used aggregated data over large geographical regions (entire provinces or territories) [7, 14, 26, 34]. Although this work in smaller communities has advanced our understanding of food insecurity, the occurrence and determinants of food insecurity for households with children have rarely been investigated in larger northern centers, including Iqaluit (Nunavut, population: 6699 [47]) [39, 45, 48]. Large northern centres contrast with smaller communities in their rapidly developing economies and large in-migration from other Canadian communities; for instance, one-third of Iqaluit immigrants arrived during the last decade [45, 47]. Furthermore, Northern urban centers are also home to a large non-Indigenous population, while smaller communities are primarily home to Inuit residents [49]. Considering these differences, determinants of food insecurity identified through previous research in smaller communities likely differs in larger centers where several factors such as sharing networks, employment, formal education, income, culturally determined food preferences, and participation in traditional harvesting activities differ [39].

Adding to recent work focusing on food security among adults in Iqaluit [45], and responding to the gaps in the literature on food security seasonality in households with children in urban Arctic settings [50], this study identified and characterised the food consumption and the food security of households with children in Iqaluit, Nunavut, Canada. Specifically, we (1) compared food consumption patterns for households with and without children, (2) estimated and compared the prevalence of food insecurity for households with and without children, (3) compared food consumption patterns and food insecurity prevalence between seasons, and (4) identified factors associated with food insecurity in households with children in Iqaluit.

## Methods

### Study setting

**Fig. 1:**
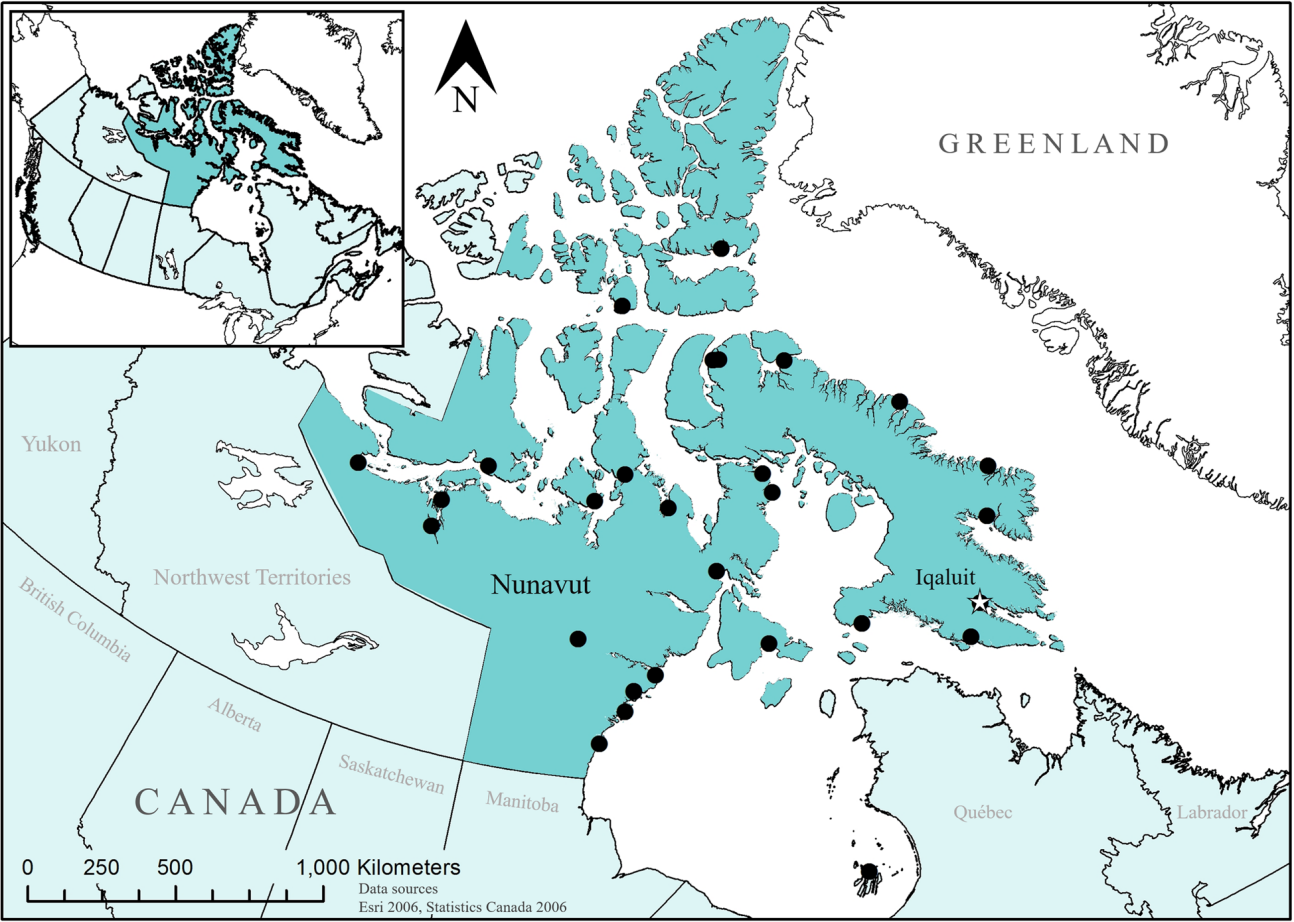
Map of Nunavut, highlighting the city of Iqaluit (*white dot*), with other communities highlighted with a *star*

Iqaluit is located on Baffin Island (63°45′N 068° 31′W), and is the capital of Nunavut with 6699 residents (Fig. 1) [47]. Iqaluit’s economy is based on waged employment from the public sector, as well as partial subsistence hunting and gathering activities. Caribou, walrus, fish, seals, beluga whales, clams, geese, and ducks are traditionally harvested and shared in the Iqaluit area [39]. A moratorium was recently imposed on caribou harvesting throughout Baffin Island, but was not in place while household surveys were conducted [51].

As the territorial capital, Iqaluit serves as the administrative centre of, and gateway community to, Nunavut and has a large and rapidly growing population (i.e. 8.3% growth rate compared to 5.9% at the national level [52]). Nearly 40% of the population identified as non-Inuit [53], reflecting a relatively recent influx of non-Indigenous people immigrating to the community. Iqaluit has a young population: in 2011, the median age was 30 years old compared to 41 years old in Canada; nearly 25% of the population in Iqaluit was aged 0 to 14 compared to less than 20% in Canada; and 60% of households had children aged 24 and under living at home compared to less than 50% in Canada [52].

### Study design

Randomly selected households were surveyed from September 15th to October 5th 2012 and from May 18th to May 31st 2013 in Iqaluit, Canada. The cross-sectional surveys were conducted during two periods to capture different harvest seasons. Although the environmental conditions that determine harvesting periods vary from year to year, late June to November is usually the ‘open water season’ in Iqaluit, during which boats are used in harvesting activities. December to the end of May typically captures ‘late fall to early spring’ during which snowmobiles are used for transportation because of generally stable ice conditions and extensive snow cover [54]. September/October and May were selected for this study as they represent the ‘shoulder seasons,’ or transition periods, during which weather fluctuations can compromise the safety of harvest activities (such as unstable ice conditions in late May, and stormy weather or the formation of land fast sea ice in October) and therefore limit access to harvesting sites and local food [54].

### Study sample

A target sample size of 512 randomly selected households was chosen to capture 25% of all households in Iqaluit (total of 2049 households) [55]. Households were selected using a two-stage random sampling protocol. This random selection was conducted in September and then households were randomly selected again in May; that is, the September and May samples were independent. First, the city was divided into 18 map components, or ‘blocks’, using the City of Iqaluit “House Numbers Atlas - February 2012” based on socioeconomic status and geographical location [55]. Blocks were selected using proportional random sampling, and then a census of the selected block was attempted; that is, every household on the block was invited to participate in the survey. Second, individuals in each household were selected to complete a two-section questionnaire: one section captured individual-level data and the other section captured household-level information. The individual-level section, relating to food consumption habits, was completed by the household member randomly selected using the ‘last birthday method’, regardless of age, in order to estimate food consumption patterns that are representative of the population [56]. The household-level section, relating to demographics, housing conditions, reliance on income support, and food security, was completed by the person in charge of household food preparation. On some occasions, the randomly selected household respondent and the person in charge of household food preparation were the same individual. All inhabitants of Iqaluit were considered potential participants for the survey, including Inuit and non-Inuit residents as well as households with and without children. Following the advice of Nunavut research partners and reflecting local research norms and expectations, respondents were compensated with a $20 CAD gift-card for local retail stores or gas stations, as well as a coupon for a larger prize draw.

Overall, 532 households completed the survey in September and 523 in May, resulting in a response rate of 75% and 55%, respectively. During the May survey, the Inuit surveyors reported that many community members were going out on the land in the weeks preceding the spring break-up, which may explain the lower response rate in May. If the household-level section was incomplete (i.e. the participant declined to complete the section or the person in charge of food preparation was unavailable), the questionnaire was not used during analysis. As a result, analyses were based on 446 questionnaires for September 2012 and 453 questionnaires for May 2013. For the purpose of this study, we grouped the households based on the presence of children (i.e. households with children and households without children). The final sample comprised 431 households with children (203 in September 2012, and 228 in May 2013) and 468 households without children (243 in September 2012, and 225 in May 2013). When compared to the 2011 Canadian Census, females, older people, and Indigenous people were over-represented in the September survey; and females and older people were over-represented in the May survey. While climate change is impacting seasonal variability in Iqaluit, our partners described the weather as “not unusual” in September 2012 and May 2013.

### Data collection

Trained members of the survey team, mainly local Inuit research assistants and some southern-based university students and academics, conducted face-to-face interviews in English, Inuktitut, or French, which represented all languages spoken by potential study participants. Some participants completed the questionnaires over the telephone at their request (9.2% in September and 17.5% in May). Questionnaires were completed using an iPad-based application (iSurvey, version 2.8.3, Wellington, New Zealand).

Food insecurity was assessed using the United States Department of Agriculture (USDA) Household Food Security Survey Module [57], which was slightly modified to reflect Inuit culture and the northern study context (questionnaire available upon request of the authors) following the Inuit Health Survey’s questionnaire (available at http://www.inuithealthsurvey.ca). A 1-month recall period was used instead of a 12-month period to allow for repeated sampling to investigate seasonal differences, as well as to increase participants’ recall reliability, which was a concern raised by local residents and decision makers during study design consultations. Ten questions were related to the food security status of adults and eight questions were related to the status of children (<18 years old) living in the household. The questionnaire was pre-tested for content and context by academics, government representatives, and community members. The USDA classification was used to determine food security status of households (see Additional files for more detail) [57]. For the purpose of this study, “*high food security*” and “*marginal food security*” were combined to indicate a food secure status; and “*low food security*” and “*very low food security*” were combined to indicate a food insecure status.

Frequency of food consumption was assessed for local foods (freshly caught fish, meat from the land, berries from the land) and retail foods (fish, meat, and pre-packaged, processed, or ready-to-eat food from the retail store). Participants reported the preparation method of local and retail fish and meat that was consumed in the past 2 weeks; these data were collected since previous studies have associated specific food preparation methods with food insecurity [25, 58–60] (see Additional files for more detail).

### Statistical analyses

Prevalence of food security status, demographic characteristics, socioeconomic characteristics, food consumption frequencies, method of food preparation characteristics, and answers to questions regarding food security were calculated. Chi-square tests were used to compare the prevalence of food insecurity between households with and without children; the prevalence of food insecurity in households with children between seasons; and the frequency of food consumption between households with and without children. To examine associations between food security status and demographic, socioeconomic, method of food preparation, and frequency of food consumption variables for households with children, we conducted a series of univariable logistic regression models for each season. All differences were considered significant at α ≤ 0.05. All statistical analyses were conducted using Stata/SE 11.2 (StataCorp LP, College Station, TX).

## Results

### Comparing food consumption patterns between households with and without children

An overview of the survey sample is provided in Table 1. As expected, households with children had significantly higher retail food expenditures, expenses related to obtaining local foods, and other household expenses compared to households without children (Table 2). In households with children, the person responsible for food preparation was more often female, Inuit, younger, and had lower formal education attainment compared to households without children (Table 2). Households with children had significantly higher levels of crowding than households without children (Table 2). There were no significant differences in employment, income support, and major household repairs needed between households with and without children (Table 2).

**Table 1:** Summary of survey participants in September 2012 and May 2013 in Iqaluit, Canada

Characteristics	Households with children			Households without children			All households
	All	September 2012	May 2013	All	September 2012	May 2013	September and May
Sample							
Total participating households (n)	488	231	257	497	259	238	985
Food security questionnaire, completed (n)	431	203	228	468	243	225	899
Age							
18–40 years old	283	132	151	192	101	91	475
41–54 years old	120	57	63	143	70	73	263
55 years old and over	28	14	14	133	72	61	161
Gender							
Female	286	141	145	275	145	130	561
Ethnic origin							
Inuit	305	149	156	237	129	108	542

**Table 2:** Prevalence of household food insecurity, demographic, and socioeconomic characteristics, September 2012 and May 2013, Iqaluit, Canada

Characteristics	Households with children				Households without children				Presence of children
	All	September 2012	May 2013	*P* value*	All	September 2012	May 2013	*P* value*	*P* value^†^
Sample									
Total participating households (n)	488	231	257		497	259	238		
Food security questionnaire, completed (n)	431	203	228		468	243	225		
Food insecurity status^a^				0.52				0.76	0.001
Food insecure^b^									
%	32.9	34.5	31.6		23.3	23.9	22.7		
95% CI	28.5, 37.4	27.9, 41.1	25.5, 37.6		19.4, 27.1	18.5, 29.3	17.2, 28.2		
Demographic characteristics									
Age^c^				0.93				0.68	<0.001
18–40 years old									
%	65.5	64.8	66.1		41.1	41.6	40.4		
95% CI	61.3, 69.7	58.6, 71.0	60.3, 72.0		36.7, 45.4	35.6, 47.7	34.1, 46.7		
41–54 years old									
%	27.9	28.3	27.6		30.5	28.8	32.3		
95% CI	23.9, 31.9	22.4, 34.1	22.1, 33.1		26.4, 35.6	23.2, 34.4	26.3, 38.3		
55 years old and over									
%	6.6	7.0	6.2		28.5	29.6	27.2		
95% CI	4.4, 8.8	3.7, 10.3	3.3, 9.2		24.5, 32.5	24.0, 35.2	21.5, 33.0		
Gender^c^				0.14				0.67	0.011
Female									
%	66.4	69.7	63.4		58.6	59.5	57.6		
95% CI	62.2, 70.6	63.7, 75.7	57.5, 69.3		54.2, 62.9	53.5, 65.5	51.3, 63.9		
Ethnic origin^d^				0.22				0.27	<0.001
Inuit									
%	70.9	73.6	68.5		50.5	52.9	47.9		
95% CI	66.9, 74.9	67.9, 79.3	62.8, 74.2		46.1, 54.9	46.8, 59.0	41.5, 54.3		
Socioeconomic characteristics									
Formal education: Incomplete secondary school^e^				0.006				0.79	0.009
%	42.4	48.9	36.6		34.3	33.7	34.9		
95% CI	38.0, 46.8	42.4, 55.4	30.7, 42.5		30.1, 38.5	27.9, 39.5	28.8, 41.0		
Employment status: Employed^f^				0.36				0.36	0.84
%	57.2	55.0	59.1		56.6	58.5	54.4		
95% CI	52.8, 61.6	48.5, 61.5	53.1, 65.2		52.2, 60.9	52.5, 64.6	48.1, 60.8		
Income support^g^				0.29				0.83	0.70
%	23.8	26.0	21.9		22.8	23.2	22.4		
95% CI	20.0, 27.6	20.3, 31.7	16.8, 27.0		19.1, 26.5	18.0, 28.3	17.0, 27.7		
Home in need of major repairs^h^				0.90				0.001	0.65
%	11.4	11.6	11.2		12.4	17.3	7.2		
95% CI	8.5, 14.3	7.4, 15.8	7.3, 15.2		9.4, 15.3	12.6, 22.0	3.9, 10.5		
Household crowding^i^				0.99				0.54	<0.001
%	17.6	17.6	17.6		2.5	3.0	2.1		
95% CI	14.1, 21.1	12.4, 22.8	12.9, 22.3		1.1, 4.0	0.8, 5.2	0.3, 3.9		
Retail food expenses^j^: Over $451 in past week				0.06				0.64	<0.001
%	32.4	36.6	28.6		12.4	13.1	11.7		
95% CI	28.2, 36.6	30.3, 42.9	23.0, 34.2		9.5, 15.4	8.9, 17.3	7.5, 15.9		
Local food expenses^k^: Over $451 in past week				0.17				0.17	0.003
%	8.3	6.7	10.8		3.1	2.1	4.9		
95% CI	5.4, 11.2	3.3, 10.1	5.6, 16.0		1.3, 4.9	0.3, 4.0	1.0, 8.7		
Other household expenses^l^: Over $1401 in past month				0.31				0.08	<0.001
%	57.7	55.2	60.0		40.4	36.6	44.8		
95% CI	53.1, 62.4	48.5, 62.0	53.6, 66.4		35.9, 45.0	30.4, 42.7	38.1, 51.5		

*P value determined using χ^2^, H_0_: Prevalence of characteristic is the same in September 2012 and May 2013

^†^ *P* value determined using χ^2^, H_0_: Prevalence of characteristic is the same in households with and without children

^a^Food secure includes high food security and marginal food security

^b^Food insecure includes low food security and very low food security

^c^Age and gender of the person responsible for food preparation

^d^Ethnic origin of the household was assumed to be the same as of the person responsible for food preparation

^e^Formal education of the person responsible for food preparation

^f^Employment status of the person responsible for food preparation. Includes part-time and full-time employment

^g^Includes income support received by any household member

^h^ “Does your home have a problem with mold or is it in need of major repairs (for example: a new roof, plumbing repairs, structural repairs)?” was asked to the household respondent

^i^Crowding is defined as “more than one person per room in the dwelling” [74]

^j^‘Retail food expenses’ include household spending in an average week for food bought from the retail store

^k^‘Local food expenses’ include household spending in an average week for obtaining or buying local food (e.g. gas, ammunition, supplies, equipment and/or local food)

^l^‘Other household expenses’ include household spending in the last month for rent, mortgage, electricity, heating fuel, gas, water and sewage, garbage, skidoo parts and oil, bullets, naphtha, and material

People in households with children consumed significantly more fruit and vegetables (*P* = 0.002) and retail foods (*P* < 0.001) compared to people in households without children (Table 3). There were no significant differences in consumption of local foods, or the frequency of consuming cooked, raw, fermented, dried or frozen fish and meats between households with and without children (Table 3).

**Table 3:** Prevalence of household food consumption characteristics, September 2012 and May 2013, Iqaluit, Canada

Characteristics	Households with children				Households without children				Presence of children
	All	September 2012	May 2013	*P* value*	All	September 2012	May 2013	*P* value*	*P* value^†^
Frequency of food consumption^a^									
Fruit and vegetables^b^									
More than half of the meals				0.35				0.73	0.002
%	79.0	80.8	77.3		70.4	69.8	71.2		
95% CI	75.3, 82.6	75.7, 85.9	72.2, 82.5		66.4, 74.5	64.1, 75.4	65.4, 77.0		
Local foods^c^									
More than half of the meals				0.19				0.08	0.20
%	11.1	13.1	9.3		8.7	10.8	6.3		
95% CI	8.3, 13.9	8.7, 17.5	5.8, 12.9		6.2, 11.2	7.0, 14.6	3.2, 9.4		
Retail foods^d^									
More than half of the meals				0.62				0.19	<0.001
%	71.4	72.5	70.4		54.9	52.1	58.0		
95% CI	67.4, 75.4	66.7, 78.3	64.8, 76.0		50.5, 59.3	46.0, 58.2	51.7, 64.3		
Method of food preparation^e^									
Cooked				0.28				0.41	0.24
%	56.6	59.2	54.3		60.3	58.5	62.2		
95% CI	52.2, 61.0	52.8, 65.6	48.2, 60.4		56.0, 64.6	52.5, 64.6	56.0, 68.4		
Raw				0.73				0.48	0.17
%	52.7	53.5	52.0		48.3	49.8	46.6		
95% CI	48.2, 57.1	47.0, 60.0	45.8, 58.1		43.9, 52.7	43.7, 55.9	40.3, 53.0		
Fermented				0.98				0.49	0.81
%	6.6	6.6	6.6		6.2	6.9	5.5		
95% CI	4.4, 8.8	3.3, 9.8	3.6, 9.7		4.1, 8.4	3.8, 10.0	2.6, 8.4		
Dried				0.003				0.09	0.42
%	43.8	50.9	37.5		41.2	44.8	37.4		
95% CI	39.4, 48.2	44.4, 57.4	31.5, 43.5		36.9, 45.6	38.7, 50.9	31.2, 43.6		
Frozen				0.98				0.42	0.20
%	61.8	61.8	61.7		57.7	59.5	55.9		
95% CI	57.4, 66.1	55.5, 68.2	55.7–67.7		53.4, 62.1	53.5, 65.5	49.5, 62.2		

^*^ *P* value determined using χ^2^, H_0_: Prevalence of characteristic is the same in September 2012 and May 2013

^†^ *P* value determined using χ^2^, H_0_: Prevalence of characteristic is the same in households with and without children

^a^Participants reported the number of meals that included these foods in the past month. For purpose of analyses, categories were combined into ‘Less than half of the meals’ (‘None’ to ‘Half of the meals’) and ‘More than half of the meals’ (‘More than half of the meals’ to ‘All meals’)

^b^Fruit and vegetables include fruit and vegetables coming from the land and retail store

^c^Local foods include freshly caught fish and meat from the land

^d^Retail foods include fish from the retail store, meat from the retail store, and pre-packaged, processed, or ready-to-eat food from the retail store

^e^Method of preparation for fish and meat, except for ‘cooked’ which includes only fish because of not enough observations for cooked meat. Participants reported consumption within the 2 weeks (yes/no)

### Comparing food security prevalence between households with and without children

**Fig. 2:**
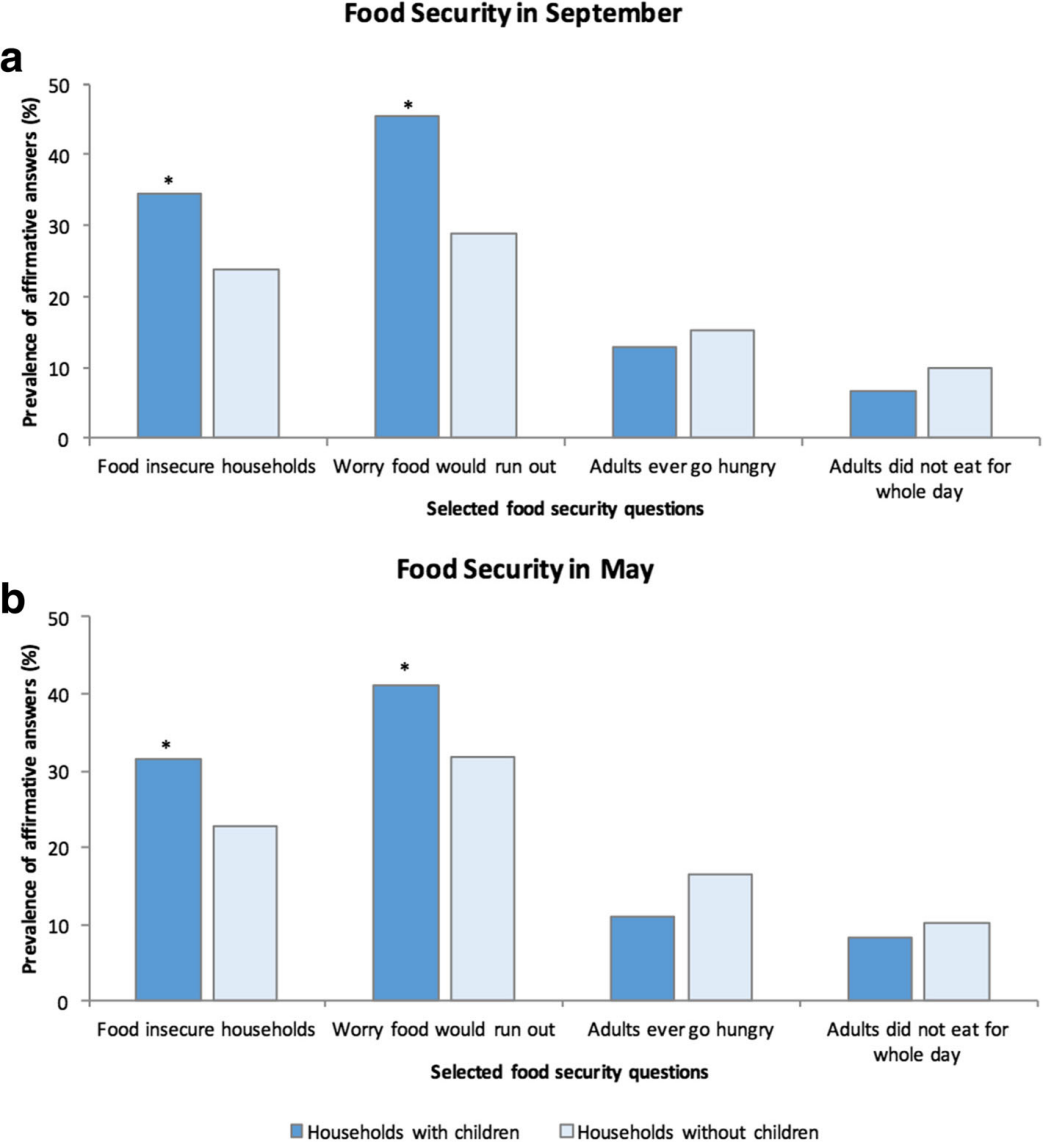
Prevalence of affirmative answers to selected food insecurity questions, Iqaluit, Canada. **a** September 2012; **b** May 2013. *P* values determined using χ2 tests, H_0_: Prevalence of affirmative answers is the same in households with and without children. * *P* < 0.001

Overall, households with children were significantly more food insecure than households without children (*P* = 0.001): 32.9% of households with children and 23.3% of households without children were food insecure in the month prior to survey (Table 2). Households with children “worried about food running out” more often than households without children in September (*P* < 0.001, Fig. 2a) and May (*P* < 0.05, Fig. 2b) (Additional file 1: Table S1 and Additional file 2: Table S2, online supplementary material).

### Comparing food consumption patterns and food security prevalence between seasons

For both households with and without children, there were no significant differences in retail food expenditures, costs associated with obtaining local food, and household expenses between May and September (Table 2). Food consumption characteristics were similar for September and May; there were no significant differences in consuming fruit and vegetables, local foods, retail foods, or cooked, raw, fermented, or frozen fish and meat between May and September. Consuming dried meat, however, differed significantly between September (51%) and May (38%) in households with children (*P* = 0.003) (Table 3). For households with children, food security status did not differ significantly between seasons (*P* = 0.52, Table 2).

### Factors associated with food insecurity in households with children

For households with children, food insecurity was significantly higher where the person responsible for food preparation was female, Inuit, employed, or had lower formal educational attainment (Table 4). For households with children, food insecurity was significantly higher when people in the household consumed more retail foods, raw fish or meat, and frozen fish or meat; conversely, food insecurity was lower for households where people consumed more fruits and vegetables, and cooked fish. Factors associated with food insecurity for households without children can be found elsewhere [45].

**Table 4:** Odds ratios (OR) of food insecurity in households with children, September 2012 and May 2013, Iqaluit, Canada^a^

Characteristics	September 2012			May 2013		
	n (%)	OR	95% CI	n (%)	OR	95% CI
Demographic characteristics						
Age^b^						
0–40 years old	149 (64.8)	*ref.*		170 (66.2)	*ref.*	
41–54 years old	65 (28.3)	1.46	0.77, 2.76	71 (27.6)	1.16	0.62, 2.15
55 years old and over	16 (7.0)	1.06	0.34, 3.30	16 (6.2)	1.00	0.29, 3.43
Gender^b^						
Male	70 (30.3)	*ref.*		94 (36.6)	*ref.*	
Female	161 (69.7)	2.21*	1.13, 4.32	163 (63.4)	1.30	0.72, 2.34
Ethnic origin^c^						
Non-Inuit	61 (26.4)	*ref.*		81 (31.5)	*ref.*	
Inuit	170 (73.6)	8.89***	3.36, 23.52	176 (68.5)	21.85***	6.60, 72.38
Socioeconomic characteristics						
Formal education^d^						
Secondary school completed	118 (51.1)	*ref.*		163 (63.4)	*ref.*	
Secondary school not completed	113 (43.9)	4.29***	2.29, 8.02	94 (36.6)	3.24***	1.80, 5.82
Employment status^e^						
Unemployed	103 (45.0)	*ref.*		105 (40.9)	*ref.*	
Employed	126 (55.0)	0.11***	0.06, 0.22	152 (59.1)	0.24***	0.13, 0.43
Retail food expenses^f^						
Less than $451	144 (63.4)	*ref.*		180 (71.4)	*ref.*	
Over $451 in past week	83 (36.6)	0.80	0.43, 1.48	72 (28.6)	1.75	0.94, 3.25
Local food expenses^g^						
Less than $451	196 (93.3)	*ref.*		124 (89.2)	*ref.*	
Over $451 in past week	14 (6.7)	1.66	0.53, 5.15	15 (10.8)	0.64	0.13, 3.12
Other household expenses^h^						
Less than $1401	94 (44.8)	*ref.*		92 (40.0)	*ref.*	
Over $1401 in past month	116 (55.2)	0.18***	0.09, 0.35	138 (60.0)	0.31***	0.16, 0.57
Frequency of food consumption^i^						
Fruit and vegetables^j^						
Less than half of the meals	44 (19.2)	*ref.*		58 (22.7)	*ref.*	
More than half of the meals	185 (80.8)	0.40*	0.20, 0.80	198 (77.3)	0.45*	0.23, 0.86
Local foods^k^						
Less than half of the meals	199 (86.9)	*ref.*		233 (90.7)	*ref.*	
More than half of the meals	30 (13.1)	1.64	0.74, 3.64	24 (9.34)	2.64	0.98, 7.16
Retail foods^l^						
Less than half of the meals	63 (27.5)	*ref.*		76 (29.6)	*ref.*	
More than half of the meals	166 (72.5)	0.79	0.41, 1.50	181 (70.4)	2.13*	1.09, 4.17
Method of food preparation^m^						
Cooked fish						
No	93 (40.8)	*ref.*		117 (45.7)	*ref.*	
Yes	135 (59.2)	0.53*	0.29, 0.96	139 (54.3)	0.50*	0.28, 0.88
Raw meat and/or fish						
No	106 (46.5)	*ref.*		123 (48.1)	*ref.*	
Yes	122 (53.5)	1.65	0.91, 2.97	133 (51.9)	1.78*	1.01, 3.14
Fermented meat and/or fish						
No	213 (93.4)	*ref.*		239 (93.4)	*ref.*	
Yes	15 (6.6)	1.17	0.37, 3.73	17 (6.6)	1.11	0.36, 3.36
Dried meat and/or fish						
No	112 (49.1)	*ref.*		160 (62.5)	*ref.*	
Yes	116 (50.9)	1.06	0.59, 1.89	96 (37.5)	0.88	0.48, 1.60
Frozen meat and/or fish						
No	87 (38.2)	*ref.*		98 (38.3)	*ref.*	
Yes	141 (61.8)	2.63**	1.39, 4.98	158 (61.7)	2.04*	1.12, 3.72

^a^ *P* value determined using an univariable logistic regression model. *P < 0.05, **P < 0.01, ***P < 0.001

^b^Age and gender are those of the person responsible for food preparation

^c^Ethnic origin of the household was assumed to be the same as of the person responsible for food preparation

^d^Formal education of the person responsible for food preparation

^e^Employment status of the person responsible for food preparation. Includes part-time and full-time employment

^f^‘Retail food expenses’ include household spending in an average week for food bought from the retail store

^g^‘Local food expenses’ include household spending in an average week for obtaining or buying local food (e.g. gas, ammunition, supplies, equipment and/or local food)

^h^‘Other household expenses’ include household spending in the last month for rent, mortgage, electricity, heating fuel, gas, water and sewage, garbage, skidoo parts and oil, bullets, naphtha, and material

^i^Participants reported the number of meals that included these foods in the past month. For purpose of analyses, categories were combined into ‘Less than half of the meals’ (‘None’ to ‘Half of the meals’) and ‘More than half of the meals’ (‘More than half of the meals’ to ‘All meals’)

^j^Fruit and vegetables include fruit and vegetables coming from the land and from the store

^k^Local foods include freshly caught fish and meat from the land

^l^Retail foods include fish from the retail store, meat from the retail store and pre-packaged, processed, or ready-to-eat food from the retail store

^m^Method of preparation includes preparation of fish and meat, except for ‘cooked’ which includes only cooked fish because of not enough observations for cooked meat. Participants reported their past 2 week consumption

## Discussion

This study compared food security and food consumption patterns between households with and without children, compared food security and food consumption patterns between seasons, as well as identified factors associated with food insecurity for households with children in Iqaluit, Nunavut. Similar to previous studies [4, 5, 7, 50], food insecurity was more common among households with children than households without children in Iqaluit. One third of households with children in this study were food insecure, which is nearly 7-fold greater than the Canadian average for households with children [2]. Nonetheless, the prevalence of food insecurity among households with children in Iqaluit (33%) was lower compared to recent studies conducted in Kugaaruk, Nunavut (83%) [5]; 16 Nunavut communities (70%) [7]; Nunavik, Quebec (50%) [34]; and a sub-Arctic First Nations community (76%) [61]. Additionally, there was a lower overall prevalence of affirmative answers to food insecurity questions in Iqaluit as compared to regional estimates for Nunavut, Inuvialuit, and Nunatsiavut [4], and Igloolik, Nunavut [6]. However, these other studies used a 12-month recall period, which is difficult to compare to the 1-month recall period used in our study. The lower prevalence of food insecurity and affirmative answers in our study may be attributable to different demographic and socioeconomic characteristics within this city centre [45]: other studies focused primarily on small Indigenous communities or regional aggregates, whereas our study focused on the larger and more economically developed northern centre, Iqaluit.

Similar to another study conducted in Iqaluit [45], there was no seasonal difference in the prevalence of food security in households with children. A variety of factors could explain the lack of seasonal differences. First, we found that retail foods were consumed more frequently than local foods in Iqaluit, which is similar to other studies conducted in Nunavut [24, 44, 62]. This might reflect the nutrition transition taking place in many Inuit communities, in which households are increasingly relying on retail rather than local foods [11–13, 62]. While local food access is heavily influenced by environmental and climatic conditions, retail food access in northern Canadian communities is less seasonally dependent [45, 54]. As such, the increasing reliance on retail foods in Iqaluit may result in seasonality being less important for food security in Iqaluit. Second, the lack of seasonal differences in food insecurity prevalence could also reflect the access to local foods via food shipments from friends and relatives in other communities, as well as soup kitchens, community freezers, and food banks [14, 44, 63]. These alternate local food access points might help negate seasonal scarcities and could explain why food insecurity did not differ by season in households with children. Lastly, Iqaluit’s economy is primarily wage-based and less dependent on subsistence activities compared to other smaller Inuit communities, thus further decreasing the influence of climatic and seasonal conditions on household incomes and sustenance [45, 64]. Importantly, we compared two ‘shoulder seasons,’ or transition periods, during which weather fluctuations can compromise the safety of harvest activities and therefore limit access to harvesting sites and local food. While we found no significant differences in food insecurity between shoulder seasons, there could be significant differences in food insecurity between shoulder and non-shoulder seasons. As such, additional research comparing food insecurity between shoulder and non-shoulder seasons warrants further research, especially in the context of climate change.

Dried meat and fish was more often consumed in September compared to May, which likely reflects the ability to produce large quantities of dried foods during the warmer and drier summer months when meat can be laid out in the sun [65, 66]. However, there were no significant seasonal differences in other food consumption patterns in Iqaluit. This lack of seasonality in food consumption patterns is similar to results reported by other food consumption frequency studies in two First Nations communities [46] and Iqaluit [45], but differs from research by Kuhnlein et al. [11] who reported seasonality in food consumption patterns in Inuit communities across the Arctic.

Contrasting previous studies [41], local food consumption was not associated with food security status in Iqaluit. This finding could be explained by the nutrition transition reportedly taking place in Iqaluit, and suggests that local food consumption may be less important for food security in larger Inuit communities where the waged economy often dominates over subsistence livelihoods [45]. As such, food security interventions in Iqaluit should be designed differently than those in other smaller Inuit communities. For instance, food security programs in Iqaluit should consider the role of this nutrition transition in food security, and consider improving access and knowledge regarding healthy retail foods, in addition to considering increasing access to local foods [24, 39].

Consumption of retail foods was associated with decreased food insecurity in households with children, which could reflect the household’s ability to afford retail foods. However, the role of retail foods in Arctic food security is complex. In the Arctic, affordable retail foods are often non-nutrient dense, with a high refined carbohydrate, fat, and sodium content [13, 16, 67, 68]. Conversely, healthy retail foods are often expensive and of low quality and freshness due to the long and sometimes delayed transport [6, 22, 44]. Previous studies documented high levels of non-nutrient dense food consumption, especially among children and youth, in Arctic communities [5, 40, 44, 62, 68]: the Inuit Child Health Survey found that on average, 35% of surveyed children’s food energy came from non-nutrient dense foods such as chips, candy, soft drinks, and fruit juice [31]. Due to these foods’ low cost per calorie, previous research suggests that Arctic food insecure households often rely on non-nutrient dense retail foods as a coping strategy for food insecurity [13, 22, 25, 28, 32, 69]. As such, improving food insecure households’ access to adequate amounts of nutritious food will require an increase in their purchasing power, either by reducing poverty or increasing the affordability of healthy eating options [22, 50], which highlights the complex role of retail foods in alleviating Arctic food insecurity.

As reported by previous studies, the odds of household food insecurity in households with children increased if the person responsible for food preparation was female [6, 18], Inuit [39], unemployed [6, 39, 45], or had a lower level of formal education [39, 45, 70].

There are several limitations to this study. First, the United States Department of Agriculture Household Food Security Survey Module does not account for important aspects of the Inuit food system, including food sharing and reliance on harvesting practices. Similar to the Inuit Health Survey, we modified the Survey Module to attempt to capture this information; however, we acknowledge that this is difficult to quantify in a survey and likely impacts the reliability of this tool in the Arctic [71]. Furthermore, it would have been valuable to capture data on the presence of a hunter within the household or as a close relation, household income, and food sharing practices, since these characteristics have previously been associated with household food security status [3]. Second, similar to other studies [45], this study limits its seasonal comparison to two seasons: September/October, and May. For Inuit, a year encompasses six 2-months seasons [16]; it would therefore be worth exploring the impacts of these shorter seasons on food security status of Inuit households with children [39]. Additionally, we collected cross-sectional data in 2012 and 2013, and we acknowledge that seasonality of food insecurity likely varies by year. Third, we acknowledge that it is important to better differentiate the food consumption habits and food insecurity levels among Inuit and non-Inuit families with children given that their respective food systems are likely different in terms of food preferences and perceptions of food insecurity. Fourth, this study did not consider the impacts of household structure or composition on food consumption, food preparation methods, or food security status [72, 73]. Lastly, questions about frequency of food consumption and method of food preparation were asked to the randomly selected household member to attempt to understand food consumption proportions at the population level; however, in identifying risk factors for food insecurity we assumed these variables were representative at the household level.

## Conclusions

Food insecurity remains a critical issue in Iqaluit. Households with children are at a greater risk of experiencing food insecurity compared to households without: one third of households with children were food insecure in Iqaluit, which is 7-fold higher than the Canadian average [2]. Retail foods were consumed more frequently than local foods, suggesting that food security interventions should consider the affordability of healthy retail food choices, in addition to programing increasing the availability of local foods. Food consumption, preparation, and security did not differ significantly by season. Several demographic and socioeconomic characteristics were associated with food security; future policies and interventions should consider the underlying social determinants of health, such as low formal education attainment and gender, which continue to aggravate food insecurity in Iqaluit. Effectively addressing the food insecurity challenge in Iqaluit will require continued research into food insecurity risk factors and trends in order to facilitate the identification of priority policy and action areas.

## Additional files

Additional file 1: Table S1. Summary of Food Security Response: September. Prevalence of affirmative answers to food insecurity questions, September 2012, Iqaluit, Canada. (DOCX 18 kb)

Additional file 2: Table S2. Summary of Food Security Response: May. Prevalence of affirmative answers to food security questions, May 2013, Iqaluit, Canada. (DOCX 130 kb)

## Abbreviations

CI: Confidence Interval
USDA: United States Department of Agriculture
