# Supplementary material for: Food insecurity and food consumption by season in households with children in an Arctic city: a cross-sectional study
Source: BMC Public Health. 2017 Jun 15;17:578. doi: 10.1186/s12889-017-4393-6 (PMC5472920; doi:10.1186/s12889-017-4393-6)
Supplement: Supplementary file 2 — Summary of Food Security Response: May. Prevalence of affirmative answers to food security questions, May 2013, Iqaluit, Canada. (DOCX 130 kb) [file 12889_2017_4393_MOESM2_ESM.docx]

**Supplemental Material**

Assessment of the household food security status

The USDA classification used to determine food security status involved assigning a value of either 1 (affirmative answers: “*Yes*”, “*Often*”, “*Sometimes*”, “*Almost every day of the month*”, and “*About half the days during the month*”) or 0 (negative answers: “*Never*”, “*No*”, and “*A few days during the month*”) for each response to the 18 questions ^(1, 2)^. The values were summed to determine a total household score. For households with children, 0 affirmative answer indicated ‘*high food security*’, 1-2 affirmative answers indicated “*marginal food security*”, 3-7 affirmative answers indicated “*low food security*”, and 8 or more affirmative answers indicated “*very low food security*”.

References

1. Nord M, Coleman-Jensen A, Andrews M et al. Household food security in the United States, 2009. Washington, D.C.: United States Department of Agriculture, Economic Research Service; 2010.

2. Bickel G, Nord M, Price C et al. Guide to measuring household food security, revised 2000. Alexandria, VA: Food and Nutrition Service, USDA; 2000.

Assessment of frequency of food consumption

Participants reported their frequency of consumption of fruit and vegetables (from the land and retail store), local foods (wild fish and meat from the land), and retail foods (fish and meat from the retail store and pre-packaged, processed, or ready-to-eat food from the retail store) over the past month using five descriptive categories (e.g. “*None*”, “*Less than half of the meals*”, “*Half of the meals*”, “*More than half of the meals*” and “*All meals*”). Participants reported the preparation method of land and retail fish and meat that was consumed in the past two weeks, which included the following categories: “*Cooked*”, “*Raw*”, “*Fermented*”, “*Dried*”, and “*Frozen*”.

**Additional file 1: Table S2.** Prevalence of affirmative answers^*^ to food security questions, May 2013, Iqaluit, Canada.

| Questions  “In the last month…” | Overall prevalence |  | May 2013 | | | | | | |  |  |
| --- | --- | --- | --- | --- | --- | --- | --- | --- | --- | --- | --- |
|  |  |  | Households with children | | |  | Households without children | | |  | Presence of children |
|  |  |  | All selected households | Food secure households | Food insecure households |  | All selected households | Food secure households | Food insecure households |  | P value^†^ |
| 1. Did you ever worry whether the food for you and your family would run out before you could get more? | | | | | | | | | |  |  |
|  | 36.8 (32.5-41.0) |  | 41.2 (35.2-47.3) | 7.1 (3.0-11.1) | 95.8 (91.2-1.01) |  | 31.9 (26.0-37.9) | 10.3 (5.8-14.9) | 94.1 (87.6-1.0) |  | 0.032 |
| 2. Did it happen that the food you bought/obtained didn't last enough time and you couldn't buy/obtain more? | | | | | | | | | |  |  |
|  | 31.3 (27.2-35.4) |  | 35.8 (29.9-41.7) | 4.5 (1.2-7.8) | 90.3 (83.3-97.2) |  | 26.5 (20.8-32.1) | 4.6 (1.5-7.7) | 92.2 (84.7-99.6) |  | 0.025 |
| 3. Were there times when you and your family could not afford to eat healthy food? | | | | | | | | | |  |  |
|  | 25.3 (21.4-29.1) |  | 27.6 (22.1-33.1) | 0 | 79.2 (69.7-88.7) |  | 22.7 (17.3-28.0) | 1.7 (-0.2-3.7) | 88.2 (79.3-97.2) |  | 0.21 |
| 4. Were there times when you could only feed your children less expensive/poor quality food because you were running out of money to buy food? | | | | | | | | | |  |  |
|  | 28.0 (22.5-33.5) |  | 28.0 (22.5-33.5) | 2.6 (0.1-5.1) | 84.7 (76.3-93.1) |  |  |  |  |  |  |
| 5. Were there times when it was not possible to feed the children a healthy meal because there was not enough money? | | | | | | | | | |  |  |
|  | 21.0 (16.0-26.0) |  | 21.0 (16.0-26.0) | 1.3 (-0.5-3.1) | 66.7 (55.6-77.7) |  |  |  |  |  |  |
| 6. Were there times when your children were not eating enough because you just couldn't buy/obtain enough food? | | | | | | | | | |  |  |
|  | 16.7 (12.1-21.3) |  | 16.7 (12.1-21.3) | 0 | 54.2 (42.5-65.8) |  |  |  |  |  |  |
| 7. Did you or other adults in your household ever cut the size of your meals or skip meals because there wasn't enough food at home? | | | | | | | | | |  |  |
|  | 17.2 (13.8-20.5) |  | 17.1 (12.5-21.7) | 0 | 54.2 (42.5-65.8) |  | 17.2 (12.4-22.0) | 0 | 74.5 (62.4-86.7) |  | 0.98 |
| 8. How often did this happen?^‡^ | | | | | | | | | |  |  |
|  | 4.4 (2.6-6.3) |  | 1.9 (0.2-3.6) | 0 | 6.9 (1.0-12.9) |  | 7.1 (3.9-10.4) | 0 | 33.3 (20.2-46.5) |  | 0.005 |
| 9. Did you or other adults in your household ever eat less than you felt you should because there wasn't enough money at home/enough to buy food? | | | | | | | | | |  |  |
|  | 16.2 (12.9-19.4) |  | 14.4 (10.1-18.7) | 0 | 50.0 (38.3-61.7) |  | 18.1 (13.2-23.0) | 0 | 78.4 (67.0-89.9) |  | 0.27 |
| 10. Were you or other adults in your household ever hungry but didn’t eat because there wasn't enough food at home? | | | | | | | | | |  |  |
|  | 13.5 (10.5-16.6) |  | 10.9 (7.1-14.7) | 0 | 37.5 (26.2-48.8) |  | 16.4 (11.7-21.1) | 0 | 76.5 (64.6-88.3) |  | 0.07 |
| 11. Did you or other adults in your household lose weight because you didn't have enough money for food? | | | | | | | | | |  |  |
|  | 8.3 (5.8-10.7) |  | 5.4 (2.7-8.2) | 0 | 19.4 (10.2-28.7) |  | 11.3 (7.3-15.4) | 0 | 52.9 (39.0-66.9) |  | 0.017 |
| 12. Did you or other adults in your household not eat for a whole day because there wasn’t enough money for food? | | | | | | | | | |  |  |
|  | 9.1 (6.6-11.6) |  | 8.2 (4.8-11.5) | 0 | 29.2 (18.5-39.8) |  | 10.1 (6.2-13.9) | 0 | 47.1 (33.1-61.0) |  | 0.46 |
| 13. How often did this happen?^‡^ | | | | | | | | | |  |  |
|  | 3.0 (1.5-4.5) |  | 1.6 (0.0-3.1) | 0 | 5.6 (0.2-10.9) |  | 4.6 (1.9-7.3) | 0 | 21.6 (10.1-33.0) |  | 0.047 |
| 14. Did you have to reduce your children's food portions because there wasn't enough food at home? | | | | | | | | | |  |  |
|  | 9.7 (6.1-13.4) |  | 9.7 (6.1-13.4) | 0 | 34.7 (23.6-45.9) |  |  |  |  |  |  |
| 15. Did any of your children have to skip meals because there was not enough food at home? | | | | | | | | | |  |  |
|  | 5.4 (2.7-8.2) |  | 5.4 (2.7-8.2) | 0 | 19.4 (10.2-28.7) |  |  |  |  |  |  |
| 16. How often did this happen?^‡^ | | | | | | | | | |  |  |
|  | 1.2 (-0.2-2.5) |  | 1.2 (-0.2-2.5) | 0 | 4.2 (-0.5-8.8) |  |  |  |  |  |  |
| 17. Did any of your children ever go hungry because there was no food at home? | | | | | | | | | |  |  |
|  | 7.8 (4.5-11.1) |  | 7.8 (4.5-11.1) | 0 | 27.8 (17.3-38.3) |  |  |  |  |  |  |
| 18. Did any of your children not eat for a whole day because there was no food at home? | | | | | | | | | |  |  |
|  | 4.3 (1.8-6.8) |  | 4.3 (1.8-6.8) | 0 | 15.3 (6.9-23.7) |  |  |  |  |  |  |

^*^Affirmative answers were “Yes”, “Often” and “Sometimes”.

^†^*P* value determined using χ^2^, presence of children used as a reference.

^‡^Affirmative answers were “Almost every day of the month” and “About half the days during the month”.
